# Processing of Waste from Enrichment with the Production of Cement Clinker and the Extraction of Zinc

**DOI:** 10.3390/ma15010324

**Published:** 2022-01-03

**Authors:** Alexandr Kolesnikov, Roman Fediuk, Olga Kolesnikova, Nurgali Zhanikulov, Bibol Zhakipbayev, Rasim Kuraev, Elmira Akhmetova, Aizhan Shal

**Affiliations:** 1 Department of “Life Safety and Environmental Protection” M. Auezov South Kazakhstan University, Building B, Av. Tauke Khan, 5, Shymkent 160012, Kazakhstan; shala96@rambler.ru; 2 Department of Science of Production and Innovation, M. Auezov South Kazakhstan University, Shymkent 160012, Kazakhstan; zhn94@ro.ru (N.Z.); bezh84@lenta.ru (B.Z.); rmkuraev@yandex.kz (R.K.); emia86@rambler.ru (E.A.); 3 Polytechnic Institute, Far Eastern Federal University, 690922 Vladivostok, Russia; fedyuk.rs@dvfu.ru; 4 Institute of Civil Engineering, Peter the Great St. Petersburg Polytechnic University, 195251 St. Petersburg, Russia; 5 Department of Inorganic and Technical Chemistry, Academician E.A. Buketov Karaganda University, Karaganda 100024, Kazakhstan

**Keywords:** thermodynamics, technogenic material, enrichment tailings, cement clinker, zinc metasilicate, zinc sublimates

## Abstract

This paper presents studies on the processing of enrichment tailings as a component of a raw mixture in order to obtain cement clinker, with simultaneous distillation of zinc. Thermodynamic studies were carried out in the temperature range of 600–1600 °C using the software application “HSC Chemistry 6” developed by the metallurgical company Outokumpu (Finland). As a result of the conducted studies, we found that zinc contributes to the intensification of mineral formation of cement clinker. In particular, it was found that the formation of belite is possible in the temperature range from 990.7 to 1500 °C with Gibbs energy values of −0.01 and −323.8 kJ (which is better than the standard process by −11.4 kJ), respectively; the formation of alite is possible in the temperature range from 982.9 to 1500 °C with Gibbs energy values of −0.05 and −402.1 kJ (better than the standard process by −11.4 kJ), respectively; the formation of tricalcium aluminate is thermodynamically possible in the temperature range from 600 °C at ΔG_T_^o^ = −893.8 kJ to 1500 °C at ΔG_T_^o^ = −1899.3 kJ (better than the standard process by −1570.1 kJ), respectively; and the formation of four calcium aluminoferrite is possible in the temperature range from 600 °C at ΔG_T_^o^ = −898.9 kJ to 1500 °C at ΔG_T_^o^ = −1959.3 kJ (better than the standard process by −1570.2 kJ), respectively, with simultaneous distillation of zinc into a gaseous state for its further capture.

## 1. Introduction

The mining and metallurgical industry is the leading sector of the Kazakh economy, accounting for 15.2% of the total industrial production. Many mining and metallurgical industries are city-forming, and therefore the development of the mining and metallurgical complex is the development of cities, jobs for more than 200 thousand people, and the accumulation of a huge amount of waste from enrichment [1,2]. In the CIS countries, the total volume of mined mining, solid minerals are about 3.5 billion m^3^ per year, and taking into account mining and preparation and processing works—about 5 billion m^3^, that is, 1.5 billion m^3^ of rocks is mined incidentally (the bulk of which, after enrichment, is stored in dumps and tailing dumps) in order to ensure the extraction of the main minerals from the ground.

At the moment, it is obvious that the modern world, as before, continues to need resources, which in the future will have a separate place in the development of the world global economy and the economy of Kazakhstan [3,4]. However, at the same time, it is necessary to critically rethink the organization of the raw material industries, namely, to revise the approaches to the management of natural resources. It is necessary to actively introduce integrated information technology platforms for natural and artificial raw materials, as well as to significantly increase the requirements for energy efficiency and resource conservation of industrial enterprises of the chemical, energy, mining, and metallurgical industries, alongside their environmental friendliness and efficiency [1,2,5,6,7,8,9,10,11,12,13,14].

Currently, due to the decrease in valuable metals in ores and the increasing amount of refractory raw materials [15,16,17], it is economically feasible and necessary to comprehensively process both poor, substandard, and hard-to-reach mineral raw materials and technogenic, as secondary raw materials [18,19], found in particular in dumps and tailings in countries such as Kazakhstan, Uzbekistan, Russia, Finland, Poland, Ukraine, Canada, and Argentina.

One such form of waste is waste from the mining and processing and metallurgical industries—tailings from the enrichment of the Balkhash Concentration Plant (BCP) in Kazakhstan [20,21], which contain in their chemical composition a number of useful compounds, in particular, silicon oxides, aluminum, and iron, which are necessary in the production of cement clinker, as well as zinc compounds, which are some of the valuable non-ferrous metals in the metallurgical industry [22,23].

It is known that the main resources in the production of binders were previously provided by traditional mineral raw materials. In the new economic realities, this approach is qualitatively changing, and industrial wastes act as secondary raw materials [15,18,19,20,24,25,26]. Moreover, the cost of such raw materials is much lower, and the processing conditions are often simple. These features of economic development insistently require a high-quality study of all types of accumulated and unused waste.

Portland cement clinker consists of a close connection of four crystalline phases of alite, belite, tricalcium aluminate (aluminate phase), and four-calcium aluminoferite (ferrite phase). The names alite, belite, aluminate and ferrite are used to distinguish them from pure phases and to distinguish them from other ions. In addition, Portland cement clinker contains a small portion of free calcium oxide and a small amount of periclase (MgO).

Portland cement clinker is produced by the synthesis of a precisely composed raw material mixture (raw flour, wet raw mass, or raw sludge). It contains elements, usually oxides, namely, CaO, SiO_2_, A1_2_O_3_, and Fe_2_O_3_, as well as a small amount of other materials. Raw flour, wet raw mass, or raw sludge are finely ground and thoroughly mixed. Portland cement clinker is a hydraulic material that must consist of at least two-thirds of calcium silicates (3CaO∙SiO_2_ and 2CaO∙SiO_2_) after firing. The rest consists of aluminum and iron-containing clinker phases and other compounds. The mass ratio of CaO/SiO_2_ should be at least 2.0. The mass of magnesium oxide (MgO) cannot exceed 5.0% [20,21,22,23,24,25].

In studies, using the calculation of the Gibbs energy (ΔG_T_^o^), we simulated the process of simultaneous synthesis of the formation of the main minerals of cement clinker (Ca_2_SiO_4_, Ca_3_SiO_5_, 3CaO∙Al_2_O_3_, 4CaO∙Al_2_O_3_∙Fe_2_O_3_) and zinc sublimates depending on the temperature from limestone and artificial tailings from the beneficiation of non-ferrous metals at the Balkhash dressing plant. The studies were carried out in order to study various dependencies, patterns, and mechanisms during the formation of a number of mineral compounds of cement clinker and zinc stripping.

Thus, research and scientific work aimed at reducing energy costs and unit costs of raw materials, involving technogenic raw materials in the production cycle as a secondary raw material, while reducing the harmful impact on the environment through waste processing, are relevant, new, and require a comprehensive qualitative study for their further development and implementation in production.

## 2. Materials and Methods

Thermodynamic studies were carried out using the “Thermodynamics” (Moscow, Russia) software [26] and the “HSC Chemistry 6” software complex, developed by the metallurgical company Outokumpu (Helsinki, Finland). The software package used in this work is based on the ideology of the European consortium SGTE (Scientific Group Thermodata Europe, Stockholm, Sweden), which develops, maintains, and distributes high-quality databases. The SGTE structure is represented by specialized research centers in Germany, Canada, France, Sweden, Great Britain, and the USA. The database of the software package contains information on 22,000 individual substances [27].

To calculate the thermodynamic functions characterizing an individual substance, we used the standard values of enthalpy *H*_298_; entropy *S*_298_; and polynomial coefficients *A*, *B*, *C*, and *D* stored in the database, from which the molar heat capacity was calculated at an arbitrarily specified temperature *T* in accordance with Equation (1).

The enthalpy of an individual substance at a temperature *T*, which differs from the standard one, equal to 298 K, was calculated by the formula:

(1) $$H_{T}=H_{298}+\int_{298}^{T}C_{p}dT+\sum H_{F},$$

where *H*_298_ is the enthalpy value of a given substance under standard conditions; *C_p_*—molar heat capacity; ∑*H_F_*—enthalpy of phase transitions (polymorphic transformations, melting, evaporation).

The entropy is defined as

(2) $$S_{T}=S_{298}+\int_{298}^{T}\frac{C_{p}}{T}dT+\frac{\sum H_{F}}{T}$$

where *S*_298_ is the value of the entropy of a given substance under standard conditions; *C_p_*—molar heat capacity; $\frac{\sum H_{F}}{T}$—entropy of phase transitions (polymorphic transformations, melting, evaporation).

As a mineral raw material, limestone of the Mynaral deposit was considered, containing 98.3% Ca, Si, C, and O in its elemental and chemical composition, and as technogenic raw materials, tailings of copper ores from the Balkhash concentrating plant, containing 90.43% consisting of Si, Fe, Ca, Al, and O in its composition; 7.76% of Na, Mg, S, K, and Ti; 1.37% Zn; and 0.44% Pb.

## 3. Results

As a result of the conducted thermodynamic studies, the Gibbs energy (ΔG_T_^o^) was calculated under conditions of modeling the synthesis of the formation of the main minerals of cement clinker (Ca_2_SiO_4_, Ca_3_SiO_5_, 3CaO∙Al_2_O_3_, 4CaO∙Al_2_O_3_∙Fe_2_O_3_) and zinc sublimates (Zn) in the temperature range 600–1600 °C. Limestone (containing mainly calcium carbonate) and technogenic (tailings from the enrichment of non-ferrous metals containing such compounds as ZnO∙SiO_2_ [21]) were the raw materials, considered groups of chemical reactions. In particular, group I—standard reactions (3)–(6) for the formation of clinker minerals [28,29,30]:

2CaCO_3_ + SiO_2_ → Ca_2_SiO_4_ + 2CO_2_↑ (3)

3CaCO_3_ + SiO_2_ → Ca_3_SiO_5_ + 3CO_2_↑ (4)

3CaCO_3_ + Al_2_O_3_ → 3CaO∙Al_2_O_3_ + 3CO_2_↑ (5)

4CaCO_3_ + Al_2_O_3_ + Fe_2_O_3_ → 4CaO∙Al_2_O_3_∙Fe_2_O_3_ + 4CO_2_↑ (6)

Group II is represented by non-standard reactions (7)–(10) of formation of clinker minerals in the presence of zinc metasilicate contained in the tailings:

ZnO∙SiO_2_ + 2CaCO_3_ → Ca_2_SiO_4_ + Zn↑ + 2CO_2_↑ + 0.5O_2_↑ (7)

ZnO∙SiO_2_ + 3CaCO_3_ → Ca_3_SiO_5_ + Zn↑ + 3CO_2_↑ + 0.5O_2_↑ (8)

ZnO∙SiO_2_ + 5CaCO_3_ + Al_2_O_3_ → → Ca_2_SiO_4_ + Zn↑ + 3CaO∙Al_2_O_3_ + 5CO_2_↑ + 0.5O_2_↑ (9)

ZnO∙SiO_2_ + 6CaCO_3_ + Al_2_O_3_ + Fe2O_3_→ → Ca_2_SiO_4_ + Zn↑ + 4CaO∙Al_2_O_3_∙Fe_2_O_3_ + 6CO_2_↑ + 0.5O_2_↑ (10)

**Figure 1:**
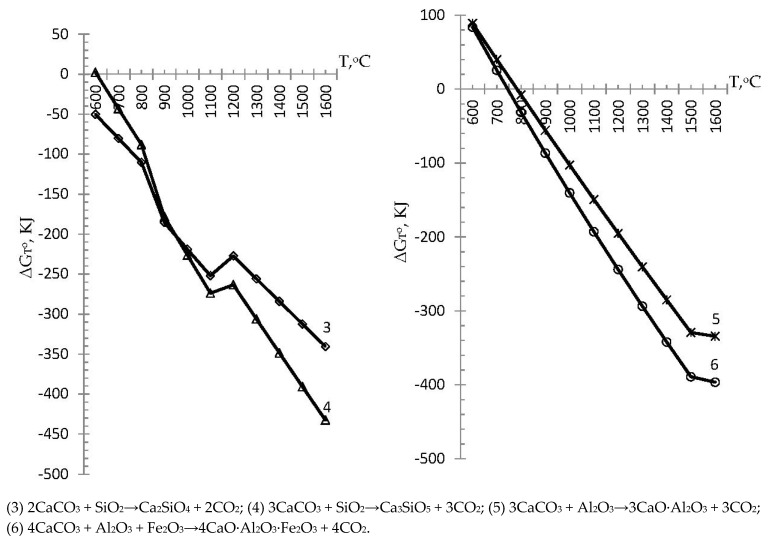
Influence of temperature on the Gibbs energy of the possibility of standard reactions with the formation of clinker minerals.

On the basis of the results of the calculations of standard reactions of group I, we plotted the dependence of the Gibbs energy on temperature (the possibility of reactions (3)–(6) with the formation of cement clinker minerals) in Figure 1. Figure 1 shows that reaction (3) with the formation of Ca_2_SiO_4_ and (4) with the formation of Ca_3_SiO_5_ proceeded practically in the entire investigated temperature range. In this case, the Gibbs energy of reaction (3) was in the negative range, which indicated the possibility of a complete reaction with the formation of belite (Ca_2_SiO_4_) within ΔG_T_^o^ from −50.2 to −340.5 kJ at 600 and 1600 °C, respectively. In contrast to reaction (3), the Gibbs energy of reaction (4) at the beginning of the investigated temperature interval had a positive value of ΔG_T_^o^ equal to 2.3 kJ at T = 600 °C, which indicates that under these conditions, the present reaction does not proceed. The onset temperature of reaction (4) was 605.2 °C when a negative value of Gibbs energy appeared, in particular, the occurrence of reaction (4) and the formation of alite (Ca_3_SiO_5_) was found to be possible in the temperature range 605.2–1600 °C with ΔG_T_^o^ from −0.006 to −432.5 kJ, respectively. At the same time, for reactions (3) and (4), the Gibbs energy of which in the temperature range of 1100–1200 °C formed a certain pronounced peak, which was explained by the polymorphism of SiO_2_. Reactions (5) and (6) shown in Figure 1 had almost the same dependence of the Gibbs energy on temperature, having almost the same positive values at 600 °C 89 and 83.9 kJ, respectively, which corresponded to their limited occurrence at the initial temperature of the research.

The Gibbs energy of reaction (5) with the possible formation of the clinker mineral 3CaO∙Al_2_O_3_ became negative at a temperature of 783.58 °C and was −0.001 kJ, reaching −334.4 kJ at T = 1600 °C. The Gibbs energy of reaction (6) with the possible formation of the clinker mineral 4CaO∙Al_2_O_3_∙Fe_2_O_3_ became negative at a temperature of 746 °C and amounted to −0.528 kJ, reaching a value of −396.5 kJ at a maximum research temperature of 1600 °C.

**Figure 2:**
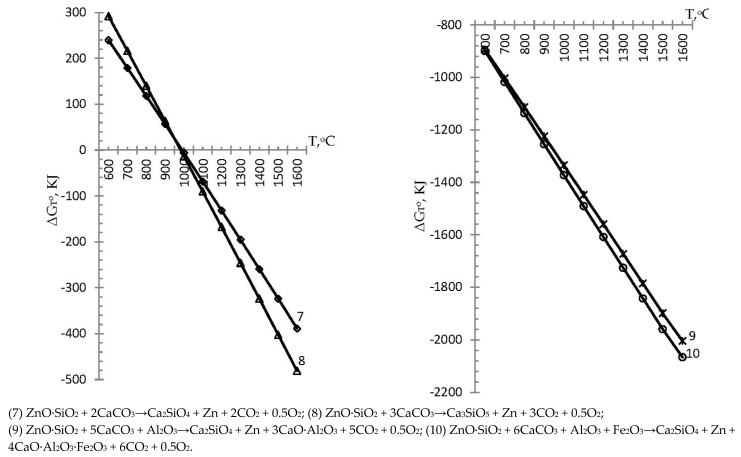
Influence of temperature on the Gibbs energy of the possibility of reactions in the presence of zinc metasilicate with the formation of clinker minerals and the simultaneous stripping of zinc.

On the basis of the results of the calculations of the reactions of group II, we plotted the graphs of the Gibbs energy dependence on temperature (the possibility of reactions (7)–(10) in the presence of zinc metasilicate with the formation of cement clinker minerals and zinc stripping) in Figure 2. Figure 2 shows that the reaction (7) with the distillation of zinc, the formation of Ca_2_SiO_4_, and (8) with the formation of Ca_3_SiO_5_ with the simultaneous distillation of zinc did not occur in the entire investigated temperature range. Thus, the Gibbs energy of reaction (7) was in the positive range at T = 600–990.6 °C, which corresponded to its impossible occurrence in this temperature range. With a further increase in temperature, the Gibbs energy of reaction (7) became negative, reaching a value of −0.015 kJ at T = 990.7 °C and becoming more negative at a maximum study temperature of 1600 °C, reaching a value of −388.6 kJ, which indicates that the reaction proceeded in the range of 990.7–1600 °C with simultaneous stripping of zinc into the gas phase and the formation of the clinker mineral belite—Ca_2_SiO_4_. Similarly to reaction (7), the Gibbs energy of reaction (8) at the beginning of the investigated temperature interval had a positive value of ΔG_T_^o^ equal to 292 kJ at T = 600 °C, which indicates that the present reaction did not proceed under these conditions.

The temperature of the onset of reaction (8) was 982.9 °C when a negative value of Gibbs energy appeared; in particular, the occurrence of reaction (8) with the transformation of zinc into a gaseous state and with the formation of the clinker mineral alite (Ca_3_SiO_5_) was possible in the temperature range of 982.9–1600 °C with ΔG_T_^o^ from −0.05 to −480.7 kJ, respectively. In this case, the Gibbs energy of reactions (7) and (8) at a temperature of 1000 °C became almost the same as the curves began to intersect, wherein the Gibbs energy of reaction (8) became more negative in contrast to reaction (7).

## 4. Discussion

From the given results of the Gibbs energy of formation according to standard reactions (3)–(6) of cement clinker minerals and according to non-standard reactions (7)–(10) with simultaneous distillation of zinc, it can be seen that both groups of reactions of formation of clinker minerals are capable of proceeding within the studied temperature. Thus, in particular, the Gibbs energy of the standard reaction (3) with the formation of belite (Ca_2_SiO_4_) had negative values in the entire investigated temperature range and ranged from −50.2 to −340.5 kJ, which indicates the possibility of the complete course of the reaction. In contrast to the standard reaction (3), the non-standard reaction (7) with the formation of Ca_2_SiO_4_ in the presence of zinc metasilicate did not occur in the entire investigated temperature range, and it was limited at the initial stage of modeling. The possibility of reaction (7) was observed at T = 990.7 °C with a negative value of the Gibbs energy (−0.015 kJ) and became more negative, reaching a value of −388.6 kJ at a maximum research temperature of 1600 °C. From this comparison, it can be seen that the standard reaction (3) had an advantage over the non-standard reaction (7) in the temperature range of 600–1400 °C; then, they changed places, and the advantage of the reaction (7) was observed at a temperature of 1500–1600 °C (with the advantage of energy values Gibbs at −11.1 and −48.1 kJ). The values of the Gibbs energy of the standard reaction (4) for the formation of alite (Ca_3_SiO_5_) at T = 600 °C had a positive value of ΔG_T_^o^ equal to 2.3 kJ and was limited. In particular, the course of reaction (4) and the formation of alite (Ca_3_SiO_5_) was possible in the temperature range 605.2–1600 °C with Gibbs energy from −0.006 to −432.5 kJ, respectively. The Gibbs energy of the non-standard reaction (8) with the formation of alite and the distillation of zinc, similarly to the standard reaction (4), was limited at the initial stage and was capable of proceeding in the temperature range 982.9–1600 °C, with a Gibbs energy from −0.05 to −480.7 kJ, respectively. Comparing the standard reaction (4) with the non-standard reaction (8), one can see the advantage of reaction (4) at a temperature of 600–1400 °C; then, at a temperature of 1500–1600 °C, the advantage of proceeding goes to a non-standard reaction (8) (with the advantage of Gibbs energy values by −11, 4, and −48.2 kJ). The Gibbs energy of the standard reaction (5) with the formation of the clinker mineral 3CaO∙Al_2_O_3_ was limited and was capable of proceeding in the temperature range 783.58–1600 °C, amounting to −0.001 kJ and −334.4 kJ, respectively. In contrast to the standard reaction (5), the non-standard reaction (9) proceeded over the entire temperature range under study, with negative Gibbs energies from −893 kJ to −20,041.1 kJ, respectively. When comparing the values of reactions (5) and (9), a significant advantage of the non-standard reaction (9) was obvious. The Gibbs energy of the standard reaction (6) was positive, which made the course of the reaction of formation of the clinker mineral 4CaO∙Al_2_O_3_∙Fe_2_O_3_ limited. Reaction (6) was capable of proceeding in the temperature range 746–1600 °C with Gibbs energies of −0.528 kJ and −396.5 kJ, respectively. Non-standard reaction (10) proceeded in the entire investigated temperature range, having negative Gibbs energies from −898.9 kJ to −20,066.2 kJ, respectively, having a significant advantage over the standard reaction (6). The data obtained were consistent with the research carried out by a number of scientists [25,26,31,32,33,34,35,36,37,38,39,40] and complement them. Regarding the distillation of non-ferrous metals, in particular, zinc, in the simulated reactions, it was shown that non-ferrous metals in the form of zinc are able to pass into the gas phase and be captured in bag filters. There are known studies by a group of authors [41,42], where experimental studies of the formation of solid solutions in zinc and lead oxides with partial substitution of non-ferrous metals of the main elements in cement clinker minerals are given. However, the manuscript considers a model with zinc metasilicate, and experimental studies of zinc behavior are envisaged in upcoming laboratory experiments.

This research will contribute to the development of a technology for the integrated processing of waste (tailings) from enrichment, as a secondary raw material with the production of cement clinker and with the simultaneous distillation and capture of non-ferrous metals in order to reduce the anthropogenic load on the natural environment of the region.

## 5. Conclusions

In group II, non-standard reactions (7)–(10) of the formation of the main minerals of cement clinker and the stripping of zinc, in particular, the occurrence of reaction (7) with the formation of belite, is possible in the temperature range from 990.7 to 1500 °C with Gibbs energies of −0.01 and −323.8kJ, respectively, better than the standard process (reaction (3)) at −11.4 kJ:

- the formation of alite during the course of reaction (8) was found to be possible in the temperature range from 982.9 to 1500 °C with Gibbs energies of −0.05 and −402.1 kJ, respectively, better than the standard process (reaction (4)) at −11.4 kJ;
- the formation of tricalcium aluminate during reaction (9) was found to be thermodynamically possible in the temperature range from 600 °C at ΔG_T_^o^ = −893.8 kJ to 1500 °C at ΔG_T_^o^ = −1899.3 kJ, better than the standard process (reaction (5)) at −1570.1 kJ;
- the formation of four calcium alumoferrite (4CaO∙Al_2_O_3_∙Fe_2_O_3_) during reaction (10) was found to be possible in the temperature range from 600 °C at ΔG_T_^o^ = −898.9 kJ to 1500 °C at ΔG_T_^o^ = −1959.3 kJ, better than the standard process (reaction (5)) at −1570.2 kJ;
- the formation of the main minerals of cement clinker in reaction group II at a temperature of 1500 °C, depending on the Gibbs energy, was found to be represented by the following series 4CaO∙Al_2_O_3_∙Fe_2_O_3_ > 3CaO∙Al_2_O_3_ > Ca_3_SiO_5_ > Ca_2_SiO_4_.

## Data Availability

Data sharing is not applicable to this article.
